# Catalase-negative *Staphylococcus aureus* isolated from a diabetic foot ulcer

**Published:** 2010-09

**Authors:** A Dezfulian, MT Salehian, V Amini, H Dabiri, M Azimirad, MM Aslani, MR Zali, I Fazel

**Affiliations:** 1 Research Center for Gastroenterology and Liver Diseases, Shahid Beheshti University of Medical Sciences, Tehran, Iran; 2 Department of General Vascular Surgery, Taleghani Hospital, Tehran, Iran; 3 Department of Microbiology, Pasteur Institute of Iran

**Keywords:** Diabetes Mellitus, Diabetic Foot, *Staphylococcus aureus*

## Abstract

We report a catalase-negative *Staphylococcus aureus* isolated from a 56-year-old male diabetic patient with foot ulcer who attended our surgery ward. Species identification was confirmed by Gram staining, standard biochemical tests and PCR amplification of the *nuc* and *fem* genes. Antibiotic susceptibility showed that the strain was sensitive to imepenem, chloramphenicol, amoxicillin, vancomycin and resistant to oxacillin, penicillin, ceftriaxone, erythromycin, clindamycin, and amikacin. Clinicians and microbiologists must be encouraged to identify and report these atypical strains and the infections associated with them in order to establish their role in pathogenesis.

## INTRODUCTION

*Staphylococcus aureus* is a gram positive, catalase and coagulase positive coccus and by far the most important pathogen among the staphylococci. It produces enzymes such as catalase which are considered to be virulence determinants. This enzyme allows bacteria to better resist intraand extra-cellular killing by hydrogen peroxide (1). Species of the genus *Staphylococcus* are characterized by the production of catalase. Among them, only two species, *Staphylococcus saccharolyticus* and *Staphylococcus aureus* subsp. *anaerobius*, are not able to produce catalase (2, 3). Although it is well known that nearly all strains of *S. aureus* have catalase activity, isolation of catalase negative Staphylococci has rarely been reported and the true incidence is not known. In routine diagnostic laboratories, catalase determination on typical strains is often not performed prior to coagulase testing, and catalase negative organisms are usually considered to be streptococci. In this study we report a case of diabetic foot caused by a catalase-negative *Staphylococcus aureus.*

## CASE REPORT

A 56-year-old male with diabetes mellitus was admitted to the surgery ward due to foot infection. After debridement of an abscess, the discharge was sent to the microbiology laboratory for culture.

Direct microscopic examination of the purulent material showed leukocytes and gram-positive cocci. Culture on 5% sheep blood agar after overnight incubation yielded smooth, raised, glistening, gray-white, beta-hemolytic colonies. The Gram-stained smear of the colonies revealed gram positive cocci in clusters. The catalase test performed with 3% H_2_ O_2_ on a glass slide was repeatedly negative. Despite the catalase negativity, coagulase production was tested by a tube coagulase test and DNAase test was done on DNAase medium and were positive, identifying the organism as *S. aureus*. The present strain differs from *Staphylococcus saccharolyticus* and *Staphylococcus aureus subsp. anaerobius* by its production of clumping factor, acid production from trehalose, mannose, and lactose and nitrate reduction (4).

The identification of the isolate as *S. aureus* subsp. *aureus* was confirmed by PCR amplification of the *nuc* and *fem* genes (5). To identify the mechanism responsible for lack of catalase activity, the nucleotide sequence of the *S. aureus* catalase gene in the catalase-negative strain was amplified by PCR using a set of primers, Cat1 5'TATAAATTGTGGAGGGATGAT3’ and Cat 2 5'TCATAAACTGCTCAACTACGC3’ (3).

Total DNA from *S. aureus* was extracted by the cetyl trimethyl ammonium bromide method after pretreatment of bacteria with lysostaphin (1 mg ml^−1^) for 1 h at 37°C in Tris/EDTA/sucrose. PCR was carried out in a 50 µl volume containing 50 ng genomic DNA with reagents and protocols supplied by the manufacturer (Roche, Germany). Thermo cycler reaction conditions were 1 min. at 94°C, 1 min. at 52°C and 1 or 1.5 min at 72°C for 30 cycles. All PCR amplifications included preliminary denaturation at 94°C for 10 min and a final incubation at 72°C for 10 min. Amplified PCR products were analysed by electrophoresis on 1% agarose gels. PCR result confirmed that this isolate is a catalase-negative *S. aureus* strain.

The susceptibility of isolate to antibiotics was determined using disk diffusion method according to CLSI (Clinical and Laboratory Standards Institute) guidelines (6). The strain was found to be sensitive to imipenem, chloramphenicol, amoxicillin, vancomycin and resistant to oxacillin, penicillin, ceftriaxone, erythromycin, clindamycin, and amikacin.

Isolates of catalase-negative *S. aureus* are extremely rare. Only a few catalase-negative *S. aureus* strains have been reported (4, 7). Catalase is a heme protein enzyme that decomposes hydrogen peroxide produced by phagocytes. The production of catalase does not appear to be essential for the growth of *S. aureus in vitro* and *in vivo* (4, 8) but it is a defense mechanism against destruction of the microorganism in phagocytic cells (2). On the other hand, there is good correlation between staphylococcal catalase activity and its lethality in mouse (8). This may explain the low frequency of infection caused by catalase negative *S.aureus* strains.

The clinical relevance of catalase-negative *S. aureus* strains requires further investigation. In previous reports, the catalase-negative *S. aureus* strains were isolated from blood samples, catheters, bronchial secretion samples, ulcers and other wounds associated with infections or nosocomial endemics (9–11). However, the true incidence of catalase-negative *S. aureus* is unknown because many diagnostic laboratories do not perform the catalase test but use Gram stain, colonial morphology, the coagulase test, and other biochemical tests for the identification of S. aureus. In conclusion, clinicians and microbiologists must be encouraged to identify and report these atypical strains and the infections associated with them, in order to establish their role in pathogenesis.
